# Modular Ontologies for Genetically Modified People and their Bioethical Implications

**DOI:** 10.1007/s11569-024-00459-4

**Published:** 2024-08-19

**Authors:** Derek So, Robert Sladek, Yann Joly

**Affiliations:** 1 Department of Human Genetics, Centre of Genomics and Policy, Victor Phillip Dahdaleh Institute of Genomic Medicine, McGill University, 740 Avenue du Dr Penfield, Room 5103, Montreal, QC H3A 0G1 Canada; 2 Departments of Medicine and Human Genetics, Victor Phillip Dahdaleh Institute of Genomic Medicine, McGill University, 740 Avenue du Dr. Penfield, Room 6214, Montreal, QC H3A 0G1 Canada; 3 Department of Human Genetics, Centre of Genomics and Policy, Victor Phillip Dahdaleh Institute of Genomic Medicine, McGill University, 740 Avenue du Dr. Penfield, Room 5101, Montreal, QC H3A 0G1 Canada

**Keywords:** Genome editing, Enhancement, Social psychology, Abstraction, Science fiction, Objective list theory

## Abstract

Participants in the long-running bioethical debate over human germline genetic modification (HGGM) tend to imagine future people abstractly and on the basis of conventionalized characteristics familiar from science fiction, such as intelligence, disease resistance and height. In order to distinguish these from scientifically meaningful terms like “phenotype” and “trait,” this article proposes the term “persemes” to describe the units of difference for hypothetical people. In the HGGM debate, persemes are frequently conceptualized as similar, modular entities, like building blocks to be assembled into genetically modified people. They are discussed as though they each would be chosen individually without affecting other persemes and as though they existed as components within future people rather than being imposed through social context. This modular conceptual framework appears to influence bioethical approaches to HGGM by reinforcing the idea of human capacities as natural primary goods subject to distributive justice and supporting the use of objective list theories of well-being. As a result, assumptions of modularity may limit the ability of stakeholders with other perspectives to present them in the HGGM debate. This article examines the historical trends behind the modular framework for genetically modified people, its likely psychological basis, and its philosophical ramifications.

## Introduction

Shortly before Chinese scientist He Jiankui sparked worldwide controversy and condemnation for his attempt to create HIV-resistant babies, he published an article in *The CRISPR Journal* about the ethics of human germline genetic modification (hereafter HGGM). Among other recommendations for public dialogue, He and his co-authors criticized the visual trope of “discombobulated babies” [1]. These images are familiar in both academic and public debate over HGGM ([2], p. 20, [3]). Cover artists for *The* *Spectator* and *The Economist* depicted babies with arrows marking out different parts of their bodies, connected to labels like “High IQ,” “low risk of Alzheimer’s,” and “not ginger” [4, 5]. *Time* magazine cover artist Hannah Whitaker photographed a baby fragmented kaleidoscopically into triangles [6] to convey that the genetically modified child “doesn’t add up to a continuous human form” [7]. *MIT Technology Review* used the illustration of a baby’s face made of floating bubbles, reminiscent of Salvador Dali’s painting *Galatea of the Spheres* [8]. When He Jiankui met the *Review*’s senior editor Antonio Regalado, he called the bubble image disgusting ([9], p. 199).

Why are these images predominant enough to pose concern for a scientist like He, who aimed to pioneer and commercialize HGGM? ([10], pp. 216–8). This article posits that “discombobulated babies” are visually powerful partly because of the way they signify anxieties about the ontology of genetically modified people, who are commonly discussed in terms of HGGM objectives like intelligence, height, strength, personality and the prevention of disease conditions [11]. To avoid calling these “phenotypes” or “traits,” which have technical meanings in biology and social psychology and import ontological assumptions from those fields, this article proposes the new term “persemes.” Similar to the way in which “ideologemes” describe basic units of ideological analysis and “mythemes” describe units of narrative structure, persemes are the culturally salient units we use to assign differences to hypothetical people. We anticipate that this term could be useful, not just in bioethics and in discussions of HGGM, but in any context that involves analyzing the conceptual distinctions between imagined people, from philosophical thought experiments to literary criticism to game design. Persemes may be assigned different ontologies in different contexts, but in the HGGM debate they are widely treated as modular entities akin to building blocks, which scientists could select and assemble into a future person.

This article begins with an overview of some historical and cognitive factors that helped perseme-based rhetoric to emerge and stabilize in the English-language academic debate on HGGM, including the disciplinary incentives and sense of “psychological distance” that led many stakeholders to focus on persemes over molecular changes or the life plans of genetically modified people. Next, the article outlines the modular ontology employed by participants in the HGGM debate when they treat persemes as internal, independent, and coequal entities assembled by scientists. The article proposes that this ontology predisposes many ethicists to accept objective list theories of well-being, as well as to examine questions of distributive justice regarding the “natural primary goods” needed to accomplish activities from the objective list. Finally, the article offers some potential strategies to help stakeholders imagine genetically modified people more concretely and broaden the bioethical debate on HGGM.

## Origins of Perseme Rhetoric in the Debate over Genetically Modified People

Although the idea of assembling people from individual persemes can be traced back to mythological narratives, English-language HGGM rhetoric that focuses on persemes and depicts these units of difference as ontologically modular appears to follow mostly from the Anglo-American eugenics movement. As Celeste Condit observes, early 20^th^ century eugenicists first relied on metaphors of animal breeding, treating each human's “germ-plasm” as a “mysterious whole” that corresponded to superior or inferior individuals without meaningful subdivisions or “assemblages of multiple characteristics” ([12] pp. 37, 129). These presumptions changed with the emergence of classical genetics. Botanist Wilhelm Johannsen proposed the “gene” in 1909 as a placeholder for the unknown mechanism of heredity ([13, 14], p. 141), creating a new “boundary object” ([15], pp. 220–224) whose adaptability helped geneticists secure their authority over naturalists, biostatisticians, animal breeders, and other competing disciplines [16].

Because each gene was considered the “smallest indivisible” unit of heredity [13] and because Mendelian conditions seemed most amenable to eugenic policymaking ([12], p. 44, [13]), eugenicists like Charles Davenport assumed that individual genes caused a wide variety of human traits ([17], pp. 66, 69, [18], pp. 60, 80). Davenport, who founded the Eugenics Record Office at Cold Spring Harbor Laboratory in 1910 ([17], p. 63, [19], p. 38), developed a manual called the *Trait Book* to help his volunteers gather information about physiological, mental, and social traits ([20], p. 58) as wide-ranging as beauty, Huntington’s disease, altruism, prostitution, and nine different means of suicide ([19], p. 40). Nathaniel Comfort observes that the *Trait Book* “flattened and equalized all human characters, from nature to nurture, from subjective to objective, from health to disease, and from trait to habit” ([19], pp. 40–41). Physical, mental, and social traits were treated as commensurable because all were believed to arise from genes in the same way ([21], pp. 66, 172).

American eugenicists also imagined that the genes underlying these traits acted in a modular fashion. In his 1929 report *Race Crossing in Jamaica*, Davenport claimed that interracial children might have “the long legs of the Negro and the short arms of the white, which would put them at a disadvantage in picking things off the ground” [22]. In such cases, he imagined, the disharmony between poorly-matched traits would result in “badly put together people” ([17] p. 148 [23] p. 112). Another eugenics study claimed that people with a tendency to religious excitation could pass down the gene for religious feeling and the gene for emotional instability in independent, Mendelian patterns ([24], p. 71). This insistence on 1-to-1 correspondences between a vast set of independent genes and phenotypes is sometimes called “beanbag genetics” ([25], p. 53, [26], p. 91), implying that, in the words of bioethicist Thomas H. Murray, “you add or pull out a bean and get the effect you seek” ([25], p. 53).

By the 1920s, this model of heredity was no longer popular outside of eugenics ([18], pp. 79, 121 [24], p. 145). Geneticists Rollins A. Emerson and Edward Murray East provided evidence against single-gene traits in plants as early as 1913 ([18], p.76, [27]). Lionel Penrose showed that mental disability was not associated with any single gene in 1938 ([19], p. 207, [28] p. 70), and most scientists turned away from eugenics for around a decade after World War II ([24], pp. 138, 205, [29], p. 135, [30], p. 3). By the 1960s, when molecular biologists began to enter the nascent HGGM debate ([31], pp. 55, 180, [32], p. 137), breakthroughs in the study of inborn errors of metabolism were drawing attention away from the complex traits that most interested eugenicists, and those were increasingly recognized as polygenic and multifactorial ([29], p. 141, [33]). Scholars who wished to discuss the improvement of culturally salient persemes like intelligence, aggression or morality [11] were unable to mention specific molecular changes because none had yet been identified; instead, most simply imagined that enhancement would take place using whichever targets and technologies were identified in the future.

In the 1980s and early 1990s, the scholarly composition of the contemporary HGGM debate began to solidify: as John H. Evans has demonstrated, theologians retreated and scientists continued to be actively involved in the debate, while bioethicists rose to become the most influential disciplinary group and lawyers and analytic philosophers both began to join discussions of HGGM ([31], pp. 135–136, 153, 1959–160). During this same time period, conceptual frameworks familiar from the classical genetics era returned as scientists attempted to identify candidate “genes for” many phenotypes ([34], p. 240, [35], p. 101, [36], pp. 353–4). As a result of these studies, persemes like “perfect pitch” were quickly incorporated into the HGGM debate in the mid-1990s ([37], p. 35, [38], p. 163, [39]), just as ethicists expanded their analyses from therapies to enhancements [40]. However, the most commonly mentioned persemes by this point in the debate included disease conditions, resistance to infection, longevity, intelligence, size, strength, eye color, hair color, and beauty [11]. Bioethicists discussing these persemes generally admitted that the enhancements that most interested them did not have any specific molecular basis that could be identified in genetics studies ([41], p. 55, [42], p. 80, [43], p. 101). 

Although speculative bioethics drew funding, legitimacy, and a sense of urgency from the technological advances associated with genomics and cloning, many scholars chose not to write about HGGM specifically; instead, they grouped together genetics, pharmaceuticals, and nanotechnology as alternative means of achieving the same persemes [44, 45]. Although the HGGM debate continued to include discussions of both disease and non-disease persemes, this process of combining technologies under the category of enhancement helped many scholars avoid discussing the molecular basis of the proposed modifications. Ari Schick discovered that the general keyword “enhancement’ emerged in bioethics literature in the mid-1990s, overtaking “genetic engineering” entirely in the following decade ([46], pp. 22–23). Indeed, a recent topic-modelling study by Bystranowski et al. determined that discussions of enhancement grew more between the 1970s and the present than any other topic in bioethics [47]. Scholars who promoted or criticized enhancements without specifying their molecular basis were able to remain unaccountable for the scientific accuracy of their speculations and to focus on whichever persemes they found most compelling, much as eugenicists had done in the early 20^th^ century.

Since there was no specific biological mechanism under discussion, many participants in the HGGM debate sidestepped safety and effectiveness concerns ([41], p. 56, [48–50]). They focused on problems that HGGM for therapy or enhancement might pose once tested and released to the public, such as the autonomy of children and parents, social stratification, stigma toward the disabled community, genetic essentialism and exceptionalism, and harms to human dignity and self-understanding. These concerns apply less to specific biological outcomes than to the social recognition of persemes whose cultural salience is reinforced by their centrality in the HGGM debate ([51, 52], p. 60). Indeed, it is possible that designers and prospective parents would be more interested in the idea of modified children being recognized for certain persemes than in the actual strength of scientific backing for HGGM ([53], p. 268, [54–56]), and companies marketing HGGM services could take advantage of these priorities by offering modifications with a questionable molecular basis [57].

In addition to acknowledging that commonly discussed persemes like intelligence, morality, or aging are polygenic and multifactorial when considered as biological traits, scholars involved in the HGGM debate often point out that these concepts are unspecific ([43], p. 119, [58], p. 29, 107, [59, 60], p. 172), lack empirical definitions ([43], p. 100–101, [53], p. 293), or represent sociocultural constructs ([61, 62], p. 75, [63], p. 327). Nevertheless, the bioethical literature has fostered an implicit ontology that shapes the way many participants in the debate imagine genetically modified people and approach ethical issues raised by the creation of those people. Persemes serve as efficient tools for discussing culturally salient, broadly understandable, and philosophically interesting human categories without being limited by technological details and questions of safety and feasibility.

Of course, scholars do not describe genetic modifications exclusively in terms of persemes, but also in terms of molecular changes and life plans. The molecular mode includes changes to the genome, such as inserting new genes or attempting to create the specific *CCR5*-Δ32 allele that He Jiankui tried to introduce. The life plan mode commonly includes careers like genetically modified athletes, musicians, super-soldiers and astronauts, as well as references to the servitor castes of *Brave New World*. These three modes do not correspond on a 1-to-1 basis; for example, stakeholders acknowledge that thousands of genetic variants may cause small differences in intelligence ([10], p. 47, [64], pp. 17–18), and they may treat intelligence either as a goal in its own right or as a capacity that comes together with other persemes to enable certain life plans ([65], pp. 40–41). Although the molecular, perseme, and life plan modes all appear often in the HGGM debate, imagery of genetically modified people has largely converged upon what Schick calls a “boilerplate” image of enhancement to persemes familiar from science fiction [45], which allow scholars to sidestep the complexity of genetics and social roles.

## Psychological Distance, Science Fiction, and Abstraction

A major reason that the HGGM debate focuses on persemes, besides the fact that this allows scholars to evade technological specifics in favour of broad cultural impacts, lies in the abstraction of the debate. When scholars think more abstractly, it is easier to discuss genetically modified people in terms of persemes than in terms of more concrete images like specific molecular changes or evocative life plans. This tendency to abstraction has been widely recognized by both proponents and critics of HGGM, who accuse each other of over-abstraction and call for more concrete and contextualized discussions ([49, 66], p. 61, [67, 68], p. 136). For instance, Peter Herissone-Kelly argues that participants in the debate usually adopt an “external perspective” similar to that of policymakers which prevents them from seeing features available only by “imaginatively inhabiting” the lives of genetically modified children [69].

Despite these recommendations, the debate has generally remained abstract and perseme-centred because it is inherently difficult to discuss hypothetical genetically modified people in detail. According to construal level theory, a branch of social psychology which originated in the work of Eleanor Rosch, scenarios with greater “hypothetical distance” (from the most probable events), “temporal distance” (from the present), or “social distance” (from the imaginer’s situation) are difficult to imagine concretely [70, 71]. All of these forms of psychological distance are associated with the HGGM debate because it is often framed as futuristic, affecting distant generations, and restricted to the wealthy.

The HGGM debate has always had a strong rhetorical emphasis on the long-term future [33, 72]. This is partly because genetic modifications and monitoring measures could involve decision-making generations in advance of the anticipated effects ([73–75], pp. 146–149, [76, 77]) and partly because anticipatory discussions of HGGM began many decades before the development of genome editing technologies like CRISPR ([72, 78], p. 76). Some scholars argue that this focus on the distant future makes emerging technologies seem more imminent than they actually are, drawing ethical attention away from genuine contemporary issues ([45, 79], pp. 194–195, [80–82], pp. 354, 357, [83]). Others, including both proponents and opponents of HGGM, argue that institutional bioethics is too focused on the present. They write that thinking about far-off potentialities can help us set aside contemporary cultural assumptions [50], focus on problems’ foundational ethical dimensions ([53], p. 94, [58], p. 34, [84, 85], p. 194, [86], p. 95), and anticipate issues well in advance of technological breakthroughs and over-hasty uses of HGGM ([30], p. 6, [43], p. 78, [44, 77, 84, 87–89], p. 195, [90], p. 37), rather than abdicating our decision-making responsibilities to future generations.

Some supporters of anticipatory governance have even accused scientists of projecting long timeframes for HGGM in order to avoid backlash and protect their grants ([90], pp. 33, 38, [91], p. 23, [92], p. 198), thus deferring any legal or regulatory restrictions onto unrealized technologies with the intention of renegotiating those limits as science progressed ([72, 93], p. 138, [94]). For example, scientists speaking at the 1968 U.S. Senate hearings on genetic engineering claimed that other medical inventions were more immediate and more worthy of concern than “lurid speculations” about HGGM ([90], p. 38). As a result of these incentives, the debate over genetically modified people has long been framed as a matter of the far-off future, helping it to remain abstract.

The idea of HGGM as belonging to the future can also draw attention away from relevant sources of information in the present. Although many stakeholders lack the direct experiences needed to imagine HGGM concretely ([95, 96], pp. 87–88, [97], p. 236, [98]), patients, doctors and children with firsthand knowledge of existing reproductive technologies, such as prenatal genetic testing and PGD, may have considerable insight into these ethical issues ([96], p.88). However, as Sarah Franklin and Celia Roberts observe in their ethnography *Born and Made,* the views and emotions of people who have experienced PGD “up close” tend to be drowned out by “far away” images of designer babies and the debate’s “relentless focus on the future” ([99], pp. xix, 14, 17–18, 220). In keeping with construal level theory, the abstraction of the debate primes participants to imagine distant events [71, 98] at the expense of tuning out concrete information about the present [70, 100] which might conflict with perseme-centred images of genetically modified people.

Although some concreteness could be restored to the HGGM debate through the use of detailed scenarios, scholars rarely devise futures that include developed characters or contexts ([31], p. 3, [46] pp. 309, 320, 323, [50, 67]). Even those who emphasize the social implications of HGGM rarely describe a well-defined time and place, provide more detail than a simple thought experiment, or extend the scenario beyond a paragraph ([34], pp. 205–206, [42], pp. 1–4, [64], pp. 13–16, 41–44, 115, 160–172, [84, 92], pp. 1–4, [101], pp. 109–131, [102], pp. 15–16, [103], pp. 10, 66–67, [104], pp. 151, [105], pp. 222–230, [106], p. 293, [107], p. 99, [108], pp. 92–101, [109], p. 146, [110], p. 1–2, [111, 112], pp. 1–7, 117–120, 199–203, 240–250, [113], pp. 1–9, [114, 115], pp. 40–55). The longest example is from the first five pages of Maxwell Mehlman and Jeffrey Botkin’s *Access to the Genome,* which describes the effects of peer pressure on a white, middle class couple in their thirties, ([37], pp. 1–5) similar to the stereotypical clients of fertility clinics and genetic screening technologies ([116, 117], pp. 259–60, [118], p. 61).

Instead of devising new scenarios, debate participants often reference pre-existing stories from science fiction, most commonly *Brave New World*, *Frankenstein*, and the 1997 film *Gattaca* [119, 120]. As mentioned above, science fiction has helped to codify certain persemes into the “boilerplate” image of a genetically modified person. That does not mean these texts are poorly written or that discussion of them is inherently harmful to the debate; in fact, thoughtful engagement with these narratives could substitute for direct experience of reproductive technologies by providing “thick description” [121], including the concrete detail and psychological interiority needed to contextualize more abstract ethical concerns ([118], pp. 19–20, 189, [122], p. 32, 43, [123, 124]).

However, as Jay Clayton observes, most bioethicists merely “invoke” *Brave New World* and “give no sign of ever having read any science fiction” [125]. (One exception is Mehlman’s *Transhumanist Dreams and Dystopian Nightmares*, which spends ten pages elaborating the plots of pertinent novels such as *Beggars in Spain* and *Oryx and Crake*) ([76], pp. 24–33). In many cases, science fiction scenarios are presented less as potential futures for society than as allegories [126]; they are deployed similarly to tales of hubris from myth and folklore such as Prometheus or Faust [120]. Participants in the HGGM debate generally use these references as brief “rhetorical commonplace” or “metaphorical shorthand” [124, 126], “common discursive denominators” between disciplines [127], or “metaphorical shorthand” [121, 128] for thematic framing, rather than providing rigorous analysis of the works’ context and content [123, 124, 126]. In the vast majority of texts, claims that HGGM is like *Brave New World, Frankenstein,* or *Gattaca* simply function as dead metaphors, contributing to psychological distance rather than concreteness and hence reinforcing perseme-centred rhetoric.

Many scholars argue that the HGGM debate relies on so many of the same tropes, analogies, practices of future extrapolation, and strategies of emotional engagement that it is essentially written in the mode of science fiction ([50, 118], p. 64, [125, 129]). Nevertheless, participants in the debate often deploy disciplinary hierarchies ([130], p. 51): by drawing an explicit rhetorical dichotomy between science fiction and reality ([42], p. 153, [54, 65], p. 42, [92], p. 57, [102], p. 167, [106], p. 13, [131], p. 7, [132], p. xiii, [133–135], p.195, [136, 137], p. 117, [138], p.54, [139–141], p. 233, [142], p. 164, [143, 144], p. 157, [145], p. 20); they apply the label of “science fiction” to their opponents’ ethical concerns as a way of calling them unrealistic [45, 146, 147]. In cases where fiction prefigured actual breakthroughs, they claim those scientific advances moved between the two categories ([9], p. xvii, [10], p. 2, [12], p. 109, [104], p. 152, [106], p. 223, [112], p. 91, [135], p. 194, [148], p. 137, [149], p. viii). For example, Lee Silver titled a chapter of his influential book *Remaking Eden* “From science fiction to reality” ([112], p. 91).

With a few notable exceptions such as Donna Haraway ([125, 150], pp. 207–210), few scholars involved in the HGGM debate have questioned conceptual boundaries between science fiction and fact. As a result, the rhetorical framing of the debate as science fiction [119] merely reinforces its psychological distance, ignoring the way science fiction authors use the genre to respond to rapid epistemic changes in society and culture ([123, 151], pp. xi, 5, 64, 73, 75, [152], pp. 6, 11, 14). This process further detracts from the importance of contemporary experiences and promotes abstraction in the way that participants imagine genetically modified people, making it easier to conceptualize them in terms of persemes.

## Conceptualizing Genetically Modified People

In addition to promoting the perseme mode over more concrete molecular changes or life plans, abstraction appears to have two major effects on conceptual frameworks for HGGM. The first is that it promotes psychological reliance on schemas [153, 154]. Schemas are experience-based images of an event, object, or person and the relationships among its parts ([155], p. 259, [156–158], pp. 33, 37), sometimes called “nodes,” “variables,” or “slots” ([155], p. 260). Schemas help us store and retrieve “default assumptions” when we have incomplete information [156], meaning that individual persemes can suggest broader stereotypes and stereotypes can suggest the presence of individual persemes ([153, 158], pp. 41–2, [159]). Author So previously conducted a discourse analysis examining how participants in the HGGM debate rely on slowly-shifting schemas of genetically modified people whose conventional “slots” have come to include health, intelligence, and cosmetic persemes like physique and eye or hair colour [11]. Stereotypical life plans, like enhanced athletes and musicians, are also schemas that can bring to mind particular persemes such as strength and perfect pitch.

The second major effect of abstraction on conceptual frameworks for HGGM is that it leads many scholars to employ more culturally-salient and less fine-grained persemes, by using vaguer terms like “intelligence” over concrete endophenotypes, like processing speed, or by using “moral enhancement” over specific behaviours [160]. Construal level theory can help us understand this process as well. We use “basic level” categories like “chair” or “dog” most readily in everyday life since they tell us how to interact with events, objects, and people ([161], pp. 46–52). We can also imagine narrower and more concrete “subordinate level” categories like types of chairs or dog breeds that may be hard to distinguish from each other, or we can imagine broader and more abstract “superordinate level” categories like “furniture” or “mammals” that may be hard to visualize as a whole ([154, 161], pp. 52–3, [162, 163], pp. 31–32). Because items categorized at the basic level, like “chairs,” are easy to tell apart from other categories but still very similar to those within the same category, the basic level of abstraction provides us the “maximum information with the least cognitive effort” ([163], p. 28).

Social psychology experiments indicate that human categories are also imagined at different levels of abstraction. Nancy Cantor and Walter Mischel found that personality-type nouns like “bright-intelligent people” or “good Samaritans” represented superordinate categories to which middle-level stereotypes like laboratory scientists or social activists belonged [162], with Susan Andersen and Roberta Klatzky supporting their results [159]. John et al. found that personality-trait adjectives like “good” and “superior” were superordinate, “kind” and “intelligent” represented the basic level, and musical talent was subordinate [164]. These findings suggest that highly schematized life plans like athletes and musicians, and persemes like morality and intelligence, are all basic-level categories, meaning that they sit at the highest level of abstraction for which we can readily form images ([161], pp. 46–52, [163], p. 34). All of these studies examined “hierarchical” or “taxonomic” relationships, meaning that category X is a specific kind of category Y.

However, categories can also be connected by “partonomic” relationships, meaning that category X is one component of category Y [164, 165]. Superordinate categories lack parts [165], but basic level categories for events, objects, and even organisms are distinguished from each other based on their parts ([161], p. 47, [165]). These parts are defined both by perceptual salience and by the discrete functions they fulfil, such as the functions of legs and seats on chairs [165]. Persemes discussed in the HGGM debate are also broadly perceived as functional, partly due to the influence of Christopher Boorse and Norman Daniels’ theories of “normal functioning” [166, 167] on the long-debated distinctions between therapy and enhancement ([30], pp. 60–65, [61, 168], pp. 145–148, [169, 170], pp. 95–105), and partly because it is common for stakeholders to imagine genetically designed people as technical products with improved functionalities and implicit teleological purposes [83, 171]. This means it is cognitively efficient for us to conceptualize basic-level persemes as units assembled into basic-level stereotypes of people like building blocks, rather than conceptualizing persemes as superordinate categories into which those stereotypes fit.

**Fig. 1:**
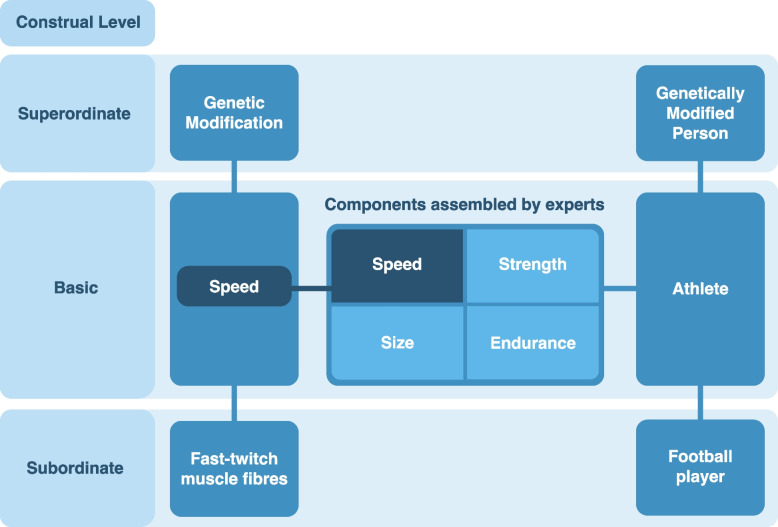
Basic-level persemes imagined as the components of genetically modified people

For example, scholars tend to discuss “speed” as a component of genetically modified athletes rather than a genetically modified athlete as a kind of fast person. Since persemes are abstracted from the molecular level and its materiality, this does not imply that many stakeholders are thinking of modified embryos as physical entities for scientists to construct. Rather, it reflects the idea of an imagined future person as a creation with components analogous to the discrete components of an object. Figure 1 shows an example of how schemas for genetically modified people and the persemes associated with them fit together in partonomic relationship.

Humans are normally more difficult to categorize than objects because social categories are more abstract, variable, and overlapping [172]. Moreover, human categories are inherently contingent on the social situations in which people apply different classifications to each other ([173], p. 312). This suggests that treating persemes as the components of future people may obscure the ways that human category labels represent comparisons drawn between people, often on the basis of unstated norms and institutional arrangements ([174], p. 49–78). Ian Hacking points out that people can respond to the labels placed upon them by changing their self-conceptions and behaviors, even in the case of “biologized kinds,” like cancer patients and alcoholics ([175], pp. 368–374). In contrast, hypothetical genetically modified people have no agency to contest our visions; like objects, they take whatever labels we assign them, making it easier for us to consider them as stereotypes ([174], p.66).

Partonomic relationships in which persemes are conceptualized as the building blocks of designed people fit easily into the HGGM debate because genetic engineering blurs psychological distinctions between the natural and artificial ([176], p. 13). In industrialized countries, genes are widely perceived as “essence placeholders,” meaning that they explain why the categories we perceive as natural are metaphysically valid ([64], p. 93, [173], p. 321, [177, 178], p. 26, [179–181]). However, philosophical arguments in the HGGM debate often posit that genetically modified people will be like manufactured artifacts in some way ([64], p. 77, [182], pp. 46–47, [183, 184], pp. 86–7, [185], pp. 453–455, [186], pp. 109–110). These associations are promoted by rhetorical conventions including references to *Brave New World* and *Frankenstein* [119, 120]*,* images of high-tech laboratory origins ([64], pp. 96–100, [99], p. 30), and metaphors like genetic “engineering” and “tinkering” ([79], p. 192, [144], p. 528, [187], p. 4, [188], p. 168, [189, 190], p. 267), which are used even by strong proponents of HGGM ([103], p. 23).

This rhetoric of artificiality may even contribute to a sense of genetic determinism by suggesting that creators’ desired functions will take precedence over the free will of the designed individuals ([64], pp. 98–100, [99], p. 33, [108], p. 53, [186], p. 109, [191, 192], p. 354), depriving them of the opportunity to define their own identities ([108], pp. 49–53, [192], p. 354). For this reason, Nadia Primc suggests that a genetically modified human should be considered a “biofact” or “living technological design” [193]. Echoing the words of Charles Davenport nearly a hundred years earlier, Kerry Lynn Mackintosh concludes that modified children might be widely viewed as “disharmonious” ([64], p. 34), especially if they have enhancements rather than therapeutic modifications which might be seen as more “natural” ([64], p. 166, [194]). Hence, it seems likely that He Jiankui, who wrote that his work might be “swallowed up by public opinion” if framed poorly to the public, [195] objected to images of “discombobulated babies” [1] at least partly because he and his collaborators worried these images would remind the public of common objections to HGGM like technological interference in the natural order, the commodification of human life, and the control of future generations.

In the following sections, we examine three major corollaries of this modular conceptual framework for genetically modified people, focusing on the way it leads many scholars involved in the HGGM debate to discuss persemes as if they were independent, ontologically “flat” compared to one another, and separated from social context.

### Persemes are Depicted as Independent

The first corollary of the modular conceptual framework is that it leads participants in the HGGM debate to imagine a genetically modified person’s persemes as independent and easily separable from one another. In the words of Owen D. Jones, these ontological assumptions represent “a paradigm shift, from baby as indivisible package to baby as mix-and-match product” [196]. As a result, participants in the debate are less likely to consider the biomedical and social issues raised by interactions between multiple modifications.

When writing in the molecular mode, scholars in the HGGM debate often warn about the need to avoid side effects from genetic pleiotropy ([54, 64], pp. 18–19, [197], p. 198, [198, 199], p. 47, [200], p. 426); for example, they note that genetic variants associated with intelligence or creativity may also be implicated in psychiatric disorders ([106], pp. 287–288, [132], p. 8, [149] p. 175, [200], p. 426, [201, 202], p. 139, [203, 204]). However, scholars almost never mention risks associated with epistasis, the way in which the effects of one gene may be influenced by other genes. This suggests that perceptions of the safety risks associated with HGGM were shaped during the early decades of the debate by discussions of monogenic conditions within families. After the advent of genome-wide association studies and polygenic scores in the 2000s, scholars who write in the molecular mode increasingly discussed modifying multiple genes at the same time. Yet, rather than discussing epistasis, they usually suggested that scientists could use HGGM to introduce allele combinations too rare to be achieved through IVF and PGD, which might in theory allow larger changes to complex traits like heart disease, cancer risk or commonly discussed enhancements like intelligence ([86], p. 46, [205–207]).

When writing in the perseme mode, scholars involved in the HGGM debate tend to take a different approach: they rarely discuss molecular-mode considerations like pleiotropy, epistasis, or polygenic inheritance at all. Rather, their rhetoric evokes the “beanbag” folk biology endorsed by most laypersons in developed countries, who assume that different genes separately control different behaviours [208]. Philosophers like Kurt Bayertz and Ronald Dworkin point out that conventional images of HGGM for persemes like beauty, aggression, or musical talent imply that human characteristics can be “assembled” like individual alleles whose effects are each independent of the modified person’s other genes ([53], p. 264, [149], p. 174, [209], p. 442). Although some opponents of HGGM, like Leon Kass and Francis Fukuyama, resist the idea of persemes as modular parts [210], Alfred Nordmann argues that scientists who promote human enhancement view it akin to “commercial product development,” treating humans as a “sum of functions” in which specific features like athletic and cognitive ability “can be optimized without destabilizing the whole system” [83].

These assumptions of independence are unrealistic even if we ignore the molecular mode entirely. Most of the commonly mentioned persemes in the HGGM debate overlap in various ways, but these are obscured by the debate’s abstraction. Categories like intelligence, memory, creativity, musical talent, and resistance to mental illness may seem largely distinct at the basic level but are much more difficult to differentiate when imagining genetically modified people and their mental characteristics in fine-grained detail. Basic level perseme categories may also be linked functionally or socially in ways obscured by the modular framework. For instance, using HGGM to prevent diseases can improve cognitive function ([211], p. 225), while larger muscles may increase not just athletic prowess but perceived attractiveness.

Persemes may also be linked together if designers choose modifications that seem to fit well with an embryo’s other predicted persemes [212], or opt to introduce conventional groupings like blue eyes and blond hair ([62], p. 38, [102], p. 166, [103], p. 32, [106], p. 93, [112], p. 202, [137], pp. 22, 117, [144], p. 188, [184], p. 228, [213], p. 217, [56, 214–218], pp. 42, 105, [219, 220], p. 81). Philosophers like Jonathan Glover, Frances Kamm, and Robert Nozick suggest that prospective parents might gravitate toward similar selections due to shared values, fashion trends, and lack of imagination ([89], p. 198, [221], pp. 47–8, [222, 223], p. 242). Maxwell Mehlman adds that parents might be induced to pick similar “packages” by peer pressure, marketing, professional group recommendations, and the lack of safe or commercially viable alternatives ([76], pp. 116–117). These perseme combinations and their connection to the potential life plans of genetically modified people deserve greater consideration in the HGGM debate.

Even if designers are not interested in providing a “package,” strong preferences for persemes like biological sex or intelligence may guide other choices. Commonly discussed life plans for genetically modified people like classical musician, basketball player, and super-soldier are all gendered, as are commonly discussed persemes like compassion ([68], p. 194, [224–226], p. 96), cooperation ([224, 226] p. 96, [227], p. 111), aggression ([62] p. 38, [68] p. 194, [228], p. 95), strength ([62], p. 38, [227], pp. 111, 215), body size ([229], p. 105), longevity ([63], pp. 328–336), attractiveness [230, 231], and even disability [231]. A perseme like height may even mean different things to designers seeking it as an aspect of attractiveness or of basketball ability. However, connections between persemes are rarely discussed in the HGGM debate, suggesting that they too are obscured by the modular framework that treats persemes as the individually assembled components of future people.

A possible reason for this lack of attention to the connections between gender and other persemes is that gender is often considered less as an organizing principle for stereotyped images of future people than deterministically as one intended modification among many others. Although biological sex is far more likely to be chosen through sperm sorting or PGD [232], it is commonly mentioned as a potential target of HGGM alongside persemes like intelligence, aggression, or beauty ([43], pp. 28, 100, 113, [68], p. 194, [76], p. 18, [105], p. 13, 191, [106], p. 228, [137], pp. 9, 51, 71, [184], p. 167, [200], p. 419, [233, 234], p. 16, [235, 236], p. 228, [237], p. 33, [238], p. 104, [239], p. 119, [240, 241], p. 166). Replacing entire chromosomes would be far more difficult than making changes to selected sequences, but the HGGM debate’s lean toward the perseme mode seems to draw attention away from these molecular distinctions. The modular framework also conceals that some modifications may work differently in biologically male and female embryos ([63], p. 334, [229], p. 108), perhaps because it conceals the role of epistatic effects in HGGM. As a result, the assumption that persemes are independent neglects commercial, cultural, and scientific factors that could lead designers to select modifications in a contextually-informed manner.

### Persemes are Depicted as Flat

The second major corollary of the modular framework is that many scholars tend to treat persemes as “flat,” meaning that they are discussed as if they were all fundamentally the same sort of thing. There are few ontological differences between persemes as discussed in the HGGM debate; each is treated similarly as a potential component of future people, like the eugenic phenotypes listed in Charles Davenport’s *Trait Book *([19] pp. 40–41, [21] pp. 66, 172). This flatness minimizes the importance of differences between the most commonly discussed persemes ([242], p. 19): they may perceived as mental (like intelligence) or physical (like disease resistance) [11], quantitative (like height) or qualitative (like beauty) ([46], p. 101, [92], p. 37, [243, 244], p. 175), discrete (like handedness) or continuous (like strength) ([230], p. 105, [245], p. 52, [246], p. 15), within the normal human range (like skin colour) or beyond it (like water breathing) [65], pp. 33, 37, 41–42, [115] p. 48, [247]), increases in one direction (like longevity) or balances between extremes (like valued personalities) ([68], p. 119, [229], p. 105), deterministic (like hair color) or unpredictable (like creativity) [240, 248], or finally early in life (like musical talent) or late in life (like Alzheimer’s resistance) ([229], p. 109, [240]).

Certainly, many different stakeholders in the debate have incentives to divide persemes into different groups. But if they can be assembled as components like building blocks, then implicitly they must all ontologically relate to the genetically modified person in a similar or even a coequal way. For example, the modular framework compresses persemes along the dimension of time by suggesting that both early-manifesting and late-manifesting ones are brought together in the embryo at the moment of assembly ([46], p. 313, [49]). As a result of this temporal flatness, bioethicists often end up discussing neither the material embryo of the present nor the “self-revealing” agent that the child will become over time in response to their experiences and their environmental context; instead, they imagine a sort of atemporal future person that contains all of their persemes alongside one another [249].

This sense of general similarity is reflected in the rhetoric of recent formal statements on HGGM, which refer in the same breath to “personality traits, eye colour, gender, cognitive ability, physical strength, etc.” ([234], p. 16); “cognitive skills/IQ, a social attitude/empathy, and exceptional musical and sporting capacities” [54]; “intelligence, memory, creativity, bravery, or strength” [250]; and “muscle type, height, longevity, and intelligence” ([115], p. 6). Common images of the HGGM process literalize this “laundry list” approach to the debate ([242], p. 19): scholars describe future parents selecting their children’s persemes from a “list” ([51], p. 134, [106], p. 293, [251], p. 223), “catalogue” ([51] p. 136), “mail order catalogue” ([252], p. 28), “Sears catalogue” ([253], p. 153), or “color wheel” ([107], p. 99). Some even describe persemes as entries on a “menu” ([149], p. 10, [230] pp. 101,103, [247]) with “flavors” [222], or a set of “apps” ([106], p. 286). These rhetorical formations present diverse uses of HGGM as ontologically similar and even interchangeable; moreover, they create the impression that almost any attribute of future people might be open to manipulation in the same way.

The idea of an omni-competent designer assembling vastly different characteristics in the embryo was enabled by the historically unstable division of labour in biomedicine ([254], pp. 19–20). In the 1970s and 1980s, clinical geneticists who aimed at “territorial expansion” by redefining known diseases as genetic found themselves stymied by other medical specialists who did not wish to see their monopolies eroded ([255], pp. 150–152). When human gene therapy emerged as a new discipline, its pioneers expanded their jurisdiction more successfully by founding journals and professional societies that covered conditions as disparate as rare genetic disorders and HIV/AIDS, assuming that methodologies developed to treat one condition might be applicable to others ([256] pp. 16, 103–104). This process may have helped to enable the perceived interchangeability of persemes under the overarching category of genetic modification, even as bioethicists’ preference for discussing persemes drew focus away from the molecular basis of HGGM.

The general sense of persemes as ontologically flat does not completely preclude essentialism, the idea that certain immutable properties necessarily cause a thing to be the kind of thing it is ([161], p. 161, [173, 180]). Essentialist perceptions apply both to species membership and to many social categories [176, 257]; as discussed earlier, persons in industrialized countries tend to see essentialized categories as the surface manifestations of underlying genetic differences [176–180]. However, not all categories associated with genes are equally essentialized. Starting in the 1950s, Celeste Condit writes, “the focus on genes as pieces of a larger code made it possible to imagine undesirable features as ‘erasable,’ that is, as separable from the fundamental being of the individual” ([12], p. 129). Philosophers in the HGGM debate generally differentiate separable and inseparable differences not in the molecular mode but in the perseme mode: they consider not the amount of the genome modified ([258–260], pp. 100–101), but whether the resulting persemes appear early in life ([58], pp. 76–77, [188], p. 162, [261, 262], p. 265) and affect the modified person’s psychology ([258, 259, 261, 262], p. 261, [263, 264], p. 213). They most frequently name biological sex as an example of a modification that would change identity ([60], p. 69, [77, 102], p. 160, [182], p. 88, [258, 264], p. 213, [265]) and eye color as a modification that would not ([213], p. 220, [258, 259, 264], p. 212, [266]). These intuitions are consistent with the results of cross-cultural social psychology experiments that find gender is the most essentialized and naturalized social category [177, 257, 267].

Although this division between “essential” and “nonessential” categories does suggest ontological differences between persemes, scholars involved in the HGGM debate rarely consider more fine-grained distinctions. For instance, psychology studies have shown that the loss of valued characteristics, such as when someone changes from kind to cruel, are more likely to be seen as identity changes than when someone gains valued characteristics by changing from cruel to kind [268, 269]. To determine whether the direction of change also matters to intuitions about HGGM, we conducted a study which found that members of the public expected someone modified to be deaf, homosexual, or blond and blue-eyed to feel as though they had been changed more than someone modified to be hearing, heterosexual, or brown-haired and brown-eyed. However, making skin colour lighter or darker were expected to have the same level of influence on self-identity (So D, Sladek R, Joly Y: Perceived effects of human germline genetic modification on personal and social identity, submitted). It is possible that some of these persemes are conceptualized as fitting into different schema “slots” than the ones into which their putative opposites fit, although further research will be required to assess this. Clearly, stakeholder perceptions of the ontology of HGGM are embedded in cultural norms, but these contextual considerations can be concealed by the modular framework and the abstraction that leads us to imagine HGGM scenarios in only the most minimal detail.

### Persemes are Depicted Without Context

The third major corollary of the modular framework for HGGM is that persemes are conceptualized as components internal to the genetically modified person, rather than as connections to other people or as the products of social relationships. One way in which this tendency manifests is in scholars’ approach to family resemblances. In practice, HGGM would likely be used to ensure that future generations of a family would have similar genes or persemes to their parents or grandparents ([68], p. 192, [190] p. 11, [270]). For example, the International Society for Stem Cell Research recommends that scientists should only “change a known pathogenic genetic variant to one that is present in unaffected family members” ([271], p. 43). HGGM could also be used to match physical resemblances ([62], p. 83) or enhanced abilities ([272], p.80). F. Allan Hanson found that women seeking intelligent sperm donors did so not primarily to give their children a competitive edge but because they felt it was one of their own qualities to pass down [273]. In such cases, persemes might be valued less as a functional component of an individual than as a sentimental link between family members ([272], p.80). However, the idea of persemes as interpersonal connections or comparisons is rarely discussed in the HGGM debate.

This lack of attention to social context may be partly due to the limited set of methods and analytical frameworks applied in the HGGM debate [274] and partly because the debate’s abstraction itself leads to highly conventional event schemas ([98, 156, 275], pp. 19–24). Scholars often resort to metaphors like tinkering and engineering because we imagine a “simplified world and the prototypical events unfolding in this world” ([275], p. 30), most frequently the selection of persemes by parents and their manipulation by scientists in a way analogous to mechanics working on machines ([189, 191, 276] p. 926, [277], p. 31). These simplified worlds minimize not just the influence of the environment on multifactorial traits but the effects of economic, sociocultural, or political constraints on HGGM decisions ([67, 191, 278], p. 174–176, [279]). They also conceal the full set of heterogeneous actors who might be involved in HGGM [50, 274]. Both He Jiankui’s idolization of Robert Edwards as a society-defying pioneer ([10], p. 228, [280, 281]) and He’s own media portrayal as a hubristic rogue scientist [281, 282] suggest that HGGM is an individual skill that might take place in isolation rather than through the input of many different stakeholders.

In a provocative effort to expand this schema, bioartist Adam Zaretsky lists over thirty “parents” involved in the birth of the genetically modified babies, including He Jiankui, the hospital ethics review board, the scientific advisory board of He’s company, business partner John Zhang, the AIDS support group through which subjects were recruited, public-relations specialist Ryan Ferrell, three universities, China’s Thousand Talents university recruitment program, international investors, and the US National Academies’ Human Gene Editing Initiative [283]. Other influential stakeholders excluded from the simplified event schema for HGGM might include medical schools, conference organizers, research consortia, journal editors, patent holders, courts, regulatory bodies, politicians, funding agencies, insurance providers, fertility clinics, egg donors and surrogates, patient advocacy groups, professional organizations such as genetic counsellors, journalists, activists for or against HGGM, religious groups, peers, and family members ([76], pp. 116–117, [115], pp. 31–39, [213], p. 25, [229], pp. 106, 110, [254], p. 66, [277], p. 174–176, [282, 284, 285], p. 27–31). Although many of these groups are discussed frequently in the debate, they are rarely imagined as providing input on the persemes of future people.

Even the legalistic idea of prospective parents as a cohesive decision-making unit ignores the possibility of conflict between parents with dissimilar values and objectives ([76], p. 78, [112], p. 202, [213], p. 25, [229], pp. 106, 110, [286], p. 104, [287], p. 244). For instance, there is some evidence that fertility clients’ values toward HGGM would display gender disparities: single women and heterosexual couples report choosing sperm donors based on somewhat different criteria [288], and surveys indicate that, on average, women are less open to HGGM than men [289]. If prospective parents are unable to decide or uninterested in selecting every modification themselves, it is possible that they might rely heavily on the input of genetic counsellors, doctors, clergy providing pastoral care, friends, or family members like grandparents who may see themselves as biologically connected to the genetically modified child.

As previously discussed, any uses of HGGM in the near future are likely to be followed by a lengthy monitoring process involving many different stakeholders, including the modified people themselves. We could think of reproductive trait selection less as a single step [290] than as a long-term process of consensus-building both before and after the actual editing, carried out between a diverse group of actors whose social positions may not even lead them to perceive persemes in the same way. Yet these relationships between heterogeneous groups appear to be concealed by the abstraction of the debate and by the modular framework. As Donna Haraway has argued, genes are often treated as “fetish objects,” meaning that they are seen as controllable “things” rather than as the products of social and political interactions such as the use of conceptual models and figurative language ([291], pp. 135, 142–143, 146). Similar assumptions are evident when participants in the HGGM debate imagine persemes as decontextualized internal entities from which to assemble future people.

## Bioethical Ramifications of the Modular Framework

For over fifty years, participants in the HGGM debate have struggled to develop the broadly acceptable ethics frameworks needed to build timely and democratically inclusive policy ([44, 91], p. Xviii, [138], p. xi, [292–294]). Progress has largely come in the calculation of costs and benefits rather than in understanding key values like autonomy ([292, 295], pp. 20–24), meaning that stakeholders with different perspectives continue to have difficulty achieving constructive dialogue ([138], p. xi,[168], p. 119, [296–299]). Recent statements aimed at outlining a path forward for HGGM, such as the U.S. National Academies’ 2017 report *Human Genome Editing: Science, Ethics, and Governance,* defer key areas of concern to public debate or to scientific consensus rather than expressing hope for advances in bioethical research [55, 299–302].

Numerous explanations have been offered for scholars’ lack of progress in the HGGM debate, including unsuitable language ([67, 99], p. 2, [188], p. 71, [209], pp. 442–443, [303, 304], p. 178, [305, 306], p. 9), unclear intuitions ([39, 188], pp. 40, 175, [247]), strong emotions associated with a sense of mythic scope ([46], pp. 304–305, [95, 307], pp. 24–25, [308, 309], p. 93, [310], p. 173, [311], p. 117), the difficulty of applying common ethical theories to potential people ([53], pp. 250, 267–271, [58], p. 39, [77, 188], pp. 12, 40–41, 55, 71, [287] p. 271, [312], pp. 121–122, [313], p. 264, [314] p. 29, [315–317]), the lack of a stable standpoint from which to consider changes to human subjectivity ([12], p. 242–243, [53], pp. 127, 174, 274–276, [99], p. 32, [148], p. 138, [209], p. 442–443, [237], p. 18, [318]), and the dominance of pluralist “regulatory” bioethics aimed at practical consensus rather than substantive moral determinations ([31], pp. 14–31, 89–91, 136, [99], pp. 3–5, 12, [319, 320], p. 158). This article proposes that stakeholders’ reliance on the modular framework when imagining genetically modified people both contributes to some of these issues and adds new impediments to the debate.

Because the modular framework limits the way we construct culturally and morally salient categories for future people and their perceived components, it results in images of HGGM that embed some ethical approaches while obscuring others. In particular, the modular framework appears to promote an autonomy-centric form of objective list theory, direct our attention to capacities rather than life plans, and suggest that we view genetic modifications as natural primary goods subject to distributive justice. These tendencies can all make HGGM seem justifiable in certain situations, meaning that more skeptical stakeholders may struggle to articulate their views in the same ethical vocabulary. The resulting conceptual gap may help to explain why many participants prefer to communicate and to structure the debate around culturally resonant narratives from science fiction, myth and folklore, or religion ([53], p. 173–174, [120, 121, 126, 127], p. 121, [321]).

### Objective List Theories of Well-Being

Analyses of ethical theory in the HGGM debate often focus on the struggle between deontological and consequentialist approaches, and on how consequentialism emerged as the more popular among secular bioethicists ([31], p. 242, [83, 203, 272] pp. 9–10, [292, 322, 323]). (For an example of virtue ethics in the HGGM debate, which has been promoted less frequently outside of theological writings, see [324].) Deontological concerns about HGGM include both hubristic attitudes ([53], p. 92, [54, 325] pp. 205–8) and infringements of human nature [53] p. 92, [54, 194, 292, 314] p. 29–30, [323, 325] p. 209–211), such as reductionist or objectifying approaches that cause indignity to genetically modified people ([53], p. 92, [59], p. 42, [171, 194, 325] p. 210, [326] pp. 74–76, [327], pp. 17, 20, [328], p. 249, [329], pp. 237–238) and violations of the human genome as the common heritage of humanity [54, 292, 326], pp. 75–78, [327] pp. 17, 20). They generally fall under what John H. Evans has labelled “substantive rationality,” in contrast to the more pluralist and consequentialist “formal rationality” that emerged for regulatory purposes in the 1980s ([31], pp. 5–31, [51, 319]). Although proponents of HGGM sometimes claim to be neutral between ethical theories [39, 330], they generally benefit from the shift toward consequentialism because of its avoidance of categorical bans ([30], p. 19) and its openness to maximizing principles ([316, 331], p. 57]). As a result, the trajectory of the debate toward regulatory bioethics has favoured the use of HGGM for therapy and produced a small, but influential, group of scientists and bioethicists who insist that enhancement is consistent with the same principles ([67, 138], p.181).

The transition from substantive rationality to formal rationality had little to do with the underlying merits of deontological or consequentialist theory, neither of which presents an easily applicable philosophical framework for HGGM ([188], pp. 12, 40, [287], p. 271, [313], p. 264, [317]). Consequentialists argue that deontological claims about human nature are arbitrary and unhelpful for coming to practical consensus, and that deontology struggles to analyze interventions like HGGM that change which individuals are brought into existence ([58], p. 42, [188], p. 40, [207, 253], pp. 159–160, [314], p. 29, [316, 317]). However, consequentialist analyses can also yield counter-intuitive results depending on which theory of well-being philosophers use ([53], pp. 267–271, [188], p. 40, [316]).

In *Reasons and Persons*, Derek Parfit divided theories of well-being into the three main categories of hedonist, desire, and “objective list” theory ([332], p. 4). In the context of HGGM, hedonism (achieving pleasure) and desire fulfilment (achieving one’s goals) could both be subverted by altering human biology to create easily contented and unambitious people, as frequent references to the brainwashed underclasses of *Brave New World* remind debate participants ([221], p. 154, [316, 333, 334], pp. 93–96, [335], pp. 3–4, [336]). Objective list theory evades these problems because it holds that there are many different goods for people, that they are intrinsic rather than instrumental, and that they are good independent of our attitudes toward them ([337] p. 148]). Typical examples include love, friendship, knowledge, reason, aesthetic experience, the development of one’s abilities, achievement, virtue, religion, play, and happiness ([332], pp. 467, 499, [334] p. 89–90, [337], p. 149). Although it is less sensitive to individual differences in taste and inclination than other theories of well-being ([338], p.107), objective list theory has become the most commonly accepted approach among contemporary philosophers [339], including many of the scholars involved in the HGGM debate ([198, 219, 328], p.264–265, [334, 340]). Because objective list theory is often similar to “folk” or “common sense” ethics, it may also be more intuitively palatable to stakeholders outside of philosophy ([219, 337], p. 152, [341] p. 133).

The major issue with objective list theory is its arbitrariness: it presents us with an “unconnected heap” of goods ([337], p. 154), failing to explain why any particular set of items should be included in the list or how to weigh these against each other when they clash ([336, 337], p. 155). The best-known solution to this critique is perfectionism, a variant of objective list theory broadly associated with philosophers like Aristotle, Aquinas, Leibniz, Marx, and Nietzsche ([331], pp. 13, 142), which posits that the objective goods are connected by their origin in human nature ([331], pp. 13–14, [341], p. 124).

The problem with using human nature to derive moral injunctions for HGGM is that, due to genetic essentialism, creating new genetic sequences or importing them from other species might change what we conceive as humanness ([42], p. 87, [144], pp. 537–538, [335], p. 3), leaving us with no clear standpoint from which to judge the problem. Members of the President’s Council on Bioethics like Michael J. Sandel, Francis Fukuyama and Leon Kass have all responded by arguing that only our current, unaltered human biology has normative value ([210, 342], p. 72, [343]), while proponents of enhancement like Allen Buchanan, Arthur Caplan, and John Harris criticize this assumption [343]. Supporters of moral enhancement, for instance, write that war-mongering and extreme cruelty are distinctively human yet objectively bad ([160, 334], pp. 84–87, [341], p. 130). Many proponents of HGGM see biology as something to be improved [210, 343] in order to help future people achieve goods from objective list theory, regardless of whether or not those goods are linked to human nature.

The assumptions about genetically modified people embedded in the modular framework support the use of objective list theory in four ways. The framework 1) associates modified persemes with the achievement of the good life by genetically modified people; 2) minimizes the pertinence of those people’s attitudes and sentiments; 3) presents them as divisible into parts; and 4) suggests they may lack a fully human nature.

First, perseme-focused rhetoric implies that commonly discussed modifications such as enhancements to intelligence, morality, and athletic ability are valuable because they enable different aspects of the “good life” to be achieved more effectively ([25], p. 121, [331], p. 168). It suggests that there exists a set of multiple goods corresponding to different human capacities, rather than a single overarching good such as pleasure or the achievement of individual desires. Since only a limited number of these are commonly discussed in the HGGM debate, the modular framework may lead participants to overlook aspects of the good life that do not conform to conventional images of genetically modified people. The strong focus on intelligence enhancement throughout the debate [11] fits with the tendency of objective list theorists and perfectionists to prioritize mental characteristics over embodied ones, and rational activity over emotion ([331], pp. 13, 85, 139) Meanwhile, as Andy Miah argues, discussions of enhanced strength and speed instead of fair play or team spirit may lead us to think of athletic achievement as centred on physical superiority ([25], p. 124).

Second, the modular framework diminishes the role of personal experience in the well-being of genetically modified people. As previously discussed, if we imagine persemes such as creativity or athletic speed as functional components selected by designers, we deny the genetically modified person full agency in determining what sort of life would be best suited for them. If we also imagine that these persemes enable the achievement of the “good life,” then the modified person’s own attitudes and preferences may seem less relevant to that outcome ([337], p.156, [341], p. 131, [344], p.152), similar to the way in which both perfectionism and objective list theory centre objective rather than subjective forms of well-being. Although happiness is widely considered to be an objective good ([337], p. 149), and psychological risks would still be important for objective list theorists to consider, proponents of HGGM often argue these risks are overblown ([197], p. 5, [345]). For example, proponents argue that intelligence enhancements would not affect genetically modified people’s sense of self-identity [210]. As Daniel M. Haybron observes, concerns based on “authenticity” can be ignored in objective list theory because it suggests the same things are good for any being, regardless of who or what it is ([346], p. 37). Objective list theory affirms that having superior capacities may matter more than what we think about having those capacities.

Third, the modular framework presents humans as divisible into independent components, similar to the way in which objective list theorists conceptualize the components of the good life. Rhetoric in the HGGM debate lists potential modifications as flattened “catalogue” or “menu” options and prioritizes different persemes for discussion based on the authors’ interests, without any coherent taxonomy or hierarchy of important or plausible modifications [11]. This approach aligns with the objective list account of goods as an “unconnected heap” of individual activities and achievements rather than an account of humans as a whole ([337], pp. 154–5). In contrast, perfectionists often advocate that the elements of the good life be developed harmoniously in accordance with their roles in human nature ([331], pp. 89, 142).

Fourth, HGGM rhetoric frames the creation of genetically modified people through technological terminology like “design” and “engineering”. Opponents of HGGM often define humanness in opposition to machines, arguing that we stand out for our essential unity and our emotional and creative capabilities ([25], p. 68, [210]). Hence, discussing genetically modified people as if they were artifacts can reinforce the idea that “human nature” does not apply to them ([65], p. 97). Since both perfectionistic and deontological approaches to HGGM take humanness as a source of norms, they may struggle to provide guidance regarding individuals who are simultaneously seen as technical products. The language and imagery associated with the modular framework leaves objective list theory as a more plausible alternative to these theories.

One response to these interpretations of the HGGM debate might be that the modular framework suggests not objective list theory, but a variant of perfectionism grounded in individual nature rather than that of the entire human species ([331], p. 15, [346], p. 32, [347], pp. 54–55). However, there as several problems with this approach. As Thomas Hurka observes, the main impediment is that our genetic profiles do not give us unique “styles of acting” ([331], p. 15). HGGM could be used to make genetically modified people vastly different from each other (which might cause them to be perceived as specialized tools), but it still might not give those people distinct definitions of well-being. Even extreme examples like being able to survive underwater or in space seem to provide new opportunities for conventional achievements, like exploration, rather than entirely novel sets of goods.

Individual perfection would also have unsavoury political implications for the HGGM debate, both because emphasizing human differences could undermine social solidarity and because it could be “fooled” by modifications like those in *Brave New World.* In theory, future designers could create a genetically modified human for whom the individual objective good was to be a placid laborer, but few proponents of HGGM would favour this. Furthermore, the fact that these unique individual natures would be enabled by conscious design rather than by the gradual evolution of the human species means that they would be reliant on the diverse and ever-changing predilections of existing people, and this does not give compelling grounds for an ethical theory.

### Autonomy, Persemes, and Life Plans

The dominance of objective list theory leaves us with the significant problem of how to prioritize modifications that are associated with an “unconnected heap” of goods. Even though commonly listed goods such as knowledge are somewhat easier to compare than the specific desires of individuals ([338], pp. 107, 122), the lack of an underlying principle to justify their inclusion in an objective list means that designers may have little ethical guidance for HGGM outside of fallible intuitions and contingent cultural norms. Even distinctions between therapy and enhancement have come under attack in the 21^st^ century by disability theorists who find them discriminatory, opponents of HGGM who believe they offer little resistance to movement down the “slippery slope” to objectionable modifications, and proponents who assert that both categories equally represent improvements to the child’s well-being ([30], pp. 60–65, 85–88, [54, 60], pp.13–16, [138], p.181, [348–351]).

This leaves designers with a wide range of persemes to choose between. Many scholars speculate that PGD decisions involving many different traits could be overwhelming for parents ([37], pp. 3–5, [112], pp. 199–201, [240, 347], pp. 193–195); indeed, couples purchasing polygenic risk scores sometimes reject all of their embryos because none of the scores seem satisfactory [352, 353]. Although adding HGGM to PGD could allow designers to change some worrying sequences, this would further increase the number of persemes for which they might feel responsible ([37], pp. 4–5, [75], p. 144, [92], p. 61, [107], pp. 99–100, [137], p. 22). Sheena Iyengar imagines a scenario where parents are overwhelmed by hundreds of options per “slot,” including specific eye colours, body measurements, and levels of cheerfulness ([107], p. 99). In addition, some persemes in different conceptual “slots” might compete with each other due to biological trade-offs ([56, 76], p. 79, [132], p. xi, [354, 355], p. 63, [356], p. 114), distinct social purposes ([76], p. 79), or even affordability concerns ([251], p. 223, [357]). Thus, it seems impossible to produce any kind of practical consensus on how the vastly different persemes in a conventional “menu” ought to be ranked ([53], p. 252, [201, 212, 251], p. 206, [354, 357–359]).

Many bioethicists attempt to resolve some of this ambiguity and make comparisons easier, as well as to avoid associations with state eugenics [340], by prioritizing autonomy as the foremost element within the objective list ([54, 338], p. 107, [360]). In doing so, they extend mainstream bioethical principlism’s deference to autonomy ([30], p. 80, [31], pp. 12, 154–155, 194, [42], pp. 372–373, [67]) into the sphere of metaphysics. Roduit et al. argue that proponents of HGGM see autonomy as key to “the bioliberal idea of a human life,” echoing perfectionist theories of human nature [343]. However, in light of these scholars’ commitments to transformative uses of HGGM and their objections to perfectionism discussed above, it is more coherent to understand this approach as a form of objective list theory that holds a prominent place for autonomy among other goods ([338], p. 237–238).

Bioethicists who seek to divide genetic modifications into ethical categories often consider whether the resulting persemes promote a future person’s freedom, leave it unaffected, or restrict it. The first category includes a wide variety of persemes termed “natural primary goods,” “all-purpose goods,” or “general-purpose means,” commonly including intelligence, health, memory, impulse control, eyesight, strength, and disease resistance, which could promote “positive” freedom by helping the modified person to achieve any life plan ([42], pp. 80, 167–8, [87, 200], pp. 425–426, [201, 333, 338], pp. 50, 71–2, [360, 361], p. 208, [362], p. 57, [363–365], p. 63, [366, 367] p. 100–101). Many supporters of HGGM agree that these modifications would contribute to the well-being of the enhanced person even if unequal access results in negative outcomes for society overall; in fact, they suggest that competition might arise precisely because, all other things being equal, these persemes are rational for everybody to want ([87, 200] pp. 424–426, [201, 360, 362], pp.60–61, [366]).

The second category of persemes includes “neutral” or “contingent” distinctions between people such as eye, hair, and skin color, biological sex, and sexual orientation, which do not provide the basic capacities needed for autonomy but do not commit the modified person to specific life plans either ([87, 219, 361] p. 205, [364, 368], pp. 7, 146–147, [369]). Bioethicists disagree on whether these kinds of modifications should be permissible; some consider them as reasonable to leave to parental choice ([364, 368], pp.7, 146–147), while others observe that they might be resented by the modified people [87], make humans more homogeneous [219], stigmatize people whose characteristics fall outside of societal tastes [364], and present uncomfortable parallels with early 20^th^ century eugenic values [369].

The third category includes persemes that commit the modified person to a particular life plan, such as extreme height to guarantee a basketball career or anatomical adaptations for space travel ([87, 219, 247, 333, 362], p. 57, [363, 367], p. 101). These life plans are inherently linked to particular social, political, and economic orders, meaning that stakeholders with different ideologies may find it difficult to compare or even to understand the motives behind these uses of HGGM ([92], p. 67, [361]). Although achievement in sports or space exploration may be objective goods ([338], p. 72), bioethicists commonly draw on the work of Joel Feinberg [370] by arguing that children must be given an “open future” ([42], pp. 170–2, [54, 104], pp. 24–44, [200], p. 426, [240, 247, 249, 362], p. 54, [364, 371, 372], p. 72, [373–375], p. 65). This includes the ability for children to reject their parents’ ideals ([58], p. 106, [104], pp. 43–44, [363]) and to thrive in unanticipated environments or peer groups [359, 361]. Many scholars argue that genetically modified people should be given the widest range of choices even if this means that they are not genetically optimized for any of their possible life plans [361, 364].

The same perseme may seem to be “all-purpose,” “neutral,” or part of a “life plan” depending on the motivation behind it. For instance, some designers may value intelligence as a general aid to success in life, some may see it as a link between family members, and some may see it as the means to a particular highly esteemed career ([104], p. 37). However, scholars tend to focus on individual capacities for three reasons. First, because consequentialist perspectives are dominant in the debate, scholars prioritize interventions that seem efficacious. HGGM aimed at individual persemes may appear more likely to succeed than trying to design people who will follow different life plans or making social changes that will allow people without those persemes to succeed equally [316, 361]. Second, because the modular framework encourages us to see persemes as independent from each other, we are less likely to connect them into complex images or narratives about the lives of future people. Third, as discussed earlier, the psychological distance of the HGGM debate favors more abstract images of genetically modified people, making it easier to discuss more abstract persemes like “intelligence” than careers like “scientist”.

Abstraction also pushes discussions away from “feasibility” toward “desirability” questions, [70, 71, 100, 154, 289] meaning that participants in the HGGM debate find it comparatively harder to write about social and political context and easier to write about foundational philosophical and ideological concerns ([51], p. 140, [67, 81, 209], p. 429, [242], p. 17, [279]) about which different stakeholders are likely to conflict in unresolvable ways [298]. With the exception of posthumanist modifications that evoke long-term survival in space, through pandemics, or underwater, few scholars have considered the broader technological, cultural, economic, and environmental changes in which specific life plans for future people would be embedded ([56, 312], p. 122). This makes it difficult to evaluate the benefit of many potential designs for future people [333]; without concrete settings in which to imagine them, it is unclear how to define a superior athlete, artist, or soldier in the first place. As Kurt Bayertz observes, life plans seem increasingly arbitrary the farther into the future we imagine ([53], pp. 276, 286), leaving us with all-purpose persemes rather than life plans as the most apparent grounds for well-being.

The conceptual distinction between all-purpose goods and specific life plans suggests a “neutralist” liberal political philosophy similar to that of John Rawls ([376], pp. 108, 328), which says we should accept only genetic modifications that help people choose whichever rational life plan they prefer ([42], p. 174, [58, 85], p. 194, [200], p. 424, [219, 338], p. 71, [362], p. 53, [363, 364, 377] p. 119]). Many of the major opponents of HGGM reject political neutralism: Jürgen Habermas proposes a form of liberalism with perfectionist assumptions about the moral self-image of human nature ([82], p. 40, [378, 379], p. 100), and Michael J. Sandel rejects the idea that a political system could ever achieve neutrality towards life plans in the first place [380]. Furthermore, as Dov Fox observes, liberal policymaking cannot address Sandel’s chief concerns about the loss of unconditional love and social solidarity [303]. Yet despite criticism of the applicability of Rawlsian social contract theory to HGGM ([188], p. 40, 55, 71), alternative approaches have struggled to gain ground, suggesting that the conceptual frameworks discussed in this article may also limit the exploration of political philosophy in the debate.

### Distributive Justice and Disability

Although the modular framework for HGGM results in only limited engagement with social and economic futures, it nevertheless embeds the idea of genetic modifications as commodities within a contemporary, liberal market economy [11, 83]. Valerie Hartouni argues that liberal and humanist discourses of possessive individualism combine with genetic essentialism to produce the idea of humans as “proprietors of their own attributes and capacities” ([381], p. 127). The independence, ontological flatness and decontextualization of persemes can further imply that attributes like HIV resistance are capable of being transferred like individual “holdings” detachable from those who hold them ([338], p. 284); as Kerry Lynn Macintosh observes, discourses of genetically modified people as artifacts also suggest that they are “fungible” ([64], p. 77, 97). Rather than unique gifts embedded in specific social links like parent–child relationships, persemes become generic, alienable commodities ([297, 306] pp. 45–46, [382]), just as images like HGGM “menus” and “mail order catalogues” imply.

Proponents of HGGM often suggest that persemes be thought of as consumer products: Jonathan Anomaly argues that mass production will make HGGM technology cheaper because from the market’s perspective “There is no principled difference between automobiles, clothing, or genetic enhancement procedures” ([132], p. 52), and Robert Nozick famously proposed a “genetic supermarket” with minimal government intervention ([223], p. 315). Robert A. Wilson suggests that this reflects a neoliberal ideology in which, rather than seeking out the same biological normalcy, consumers are expected to choose from “an endless configuration of marketable traits” [20]. Whereas opponents of HGGM express concern about protecting future parents from market pressures, proponents are more likely to focus on “irrational” pressures such as tradition ([138], p.222). However, most proponents still recognize the need for regulations to prevent harm to both individuals and society as a result of these choices ([41], p.66, [160, 362], p. 51–52).

The idea that monetary value can be assigned to persemes ([52], p. 61), combined with the mildly determinist premise that therapeutic or enhancing genetic modifications might be safe and effective enough to be worth large sums [383], have led many scholars, and especially proponents of HGGM, to frame the debate as centrally a question of distributive justice ([30], pp.87–88, [103], p. 77). For example, George Church has stated that “When people talk about the ethics of CRISPR, 90 percent of it should be, and probably is, about equal distribution of expensive technology” [384]. If valued persemes are conceptualized as natural primary goods that contribute to the objective good of autonomy, then we are effectively discussing the distribution of autonomy among socioeconomic groups.

Perfectionist and objective-list theories of well-being can contribute to this ethical focus on unjust distributions of HGGM because both theories have often been criticized as elitist ([337] p,. 156, [338] p. 60, [341], pp. 132–133, [380]), evoking eugenicists’ beliefs in a biologically-justified aristocracy ([12] p. 39, [17] p. 78, 161). If the natural primary goods are what allow people to carry out objectively valuable lives, then human well-being might be maximized by giving those with greater “natural talent” more resources to support their development ([331], pp. 130, 183, [341], pp. 132–3, [357]). Expensive HGGM procedures could exacerbate inequalities even further by reducing diseases and concentrating persemes perceived as valuable in the children of the wealthy; however, some scholars argue that HGGM could also reduce the role of luck in opportunity if it were used to distribute those capacities to children who would otherwise have been genetically disadvantaged ([42], p. 76, [54, 338], p. 50, [360, 385]).

 The more scholars focus on the distribution of capacities, the more they present HGGM as a problem amenable to “regulatory” bioethics within the tradition of liberal political philosophy. From this perspective, the neutralist liberal approach represents a practical tool for resource distribution without requiring practioners to endorse any particular view on the substantive implications of HGGM for humanness ([200], p. 424). As a result, the conceptual frameworks discussed in this article also have significant consequences for the way many participants in the HGGM debate conceptualize disability. The categorization of persemes like “normal” height and hearing as natural primary goods that improve autonomy and help anyone achieve their own life plan is consistent with neutralist liberalism ([42], pp. 142, 168). If specific persemes are associated with the achievement of objective goods like athletics, people with disabilities like dwarfism will be seen as living objectively deprived lives ([20], p.157, [272], p.139, [386], p. 264). This approach hinders the use of “thicker,” more socially-sensitive conceptions of human flourishing that draw on more substantive values and lived experiences ([31], p. 201, [121, 298, 367, 387], p.21).

Disability rights advocates and philosophers critical of HGGM, like Habermas, often argue that there is nothing “neutral” about the elimination of disability ([20], p.157, [42], p. 167, [170], pp. 108, 114, [182], p.86, [219]), but many of their arguments do not mesh with the modular framework. For instance, the common “expressivist critique” of HGGM states that disabilities are always embodied in people and that preventing deafness or dwarfism sends the message to disabled people that they should not exist ([20], p. 150, [42], pp. 272–281, [86], pp. 82–83, [265, 334], pp. 34–35, [388], p.88, [389], p. 147, [390], pp. 237–240, [391], pp. 112–116). Proponents of HGGM typically respond that individual persemes like deafness or dwarfism are conceptually separable from people, meaning that people are able to wish they had been born without those conditions ([42], p. 278, [86], p. 84, [213], p. 115, [222, 229], p. 110, [272], p. 151, [389], p. 147, [391], pp. 116–118, [392]). (By the same token, future people would be able to wish they had been born with enhancements, at least for the many persemes that are insufficiently essentialized to trigger concerns about identity change.) Since persemes considered as modular components are easier to conceptualize separately from their bearers, the modular framework helps to insulate HGGM against charges of contributing to discrimination against the disabled.

## Conclusions and Future Directions

This article emerged as an effort to understand why the HGGM debate is so commonly framed with images like “discombobulated babies” and features selected off a “menu,” metaphors that present genetically modified people as divisible into distinct components. The resulting investigation combined analyses of rhetoric from over a century of eugenics and HGGM literature with theories from social psychology. Ultimately, we proposed a model of how participants in the debate tend to conceptualize hypothetical people and of how the modular conceptual framework has shaped bioethical theory.

Observing that most HGGM discourses were structured less by references to molecular changes or to the life plans of genetically modified people than to individual characteristics like intelligence, morality, and strength, we proposed “persemes” as a new general-purpose term to describe the conventional units that bioethicists and other thinkers use to assign differences to hypothetical people, avoiding confusion with scientifically meaningful terms like traits or phenotypes. Although perseme rhetoric is common among academic disciplines and laypersons, this mode of imagining future people was particularly useful both to eugenicists in the early 20^th^ century and to participants in the HGGM debate because it allowed scholars to focus on culturally and philosophically salient concerns rather than concrete biological differences. This rhetoric also benefited from the long-term timeframes and science fiction framing of the debate that led to a high level of abstraction, directing many scholars’ focus to schematized images of future people with “basic level” persemes rather than to specific behaviors, well-contextualized scenarios, or the experiences of stakeholders in the present.

Persemes in the HGGM debate are broadly discussed as if they were “building blocks” assembled within semi-artificial people rather than messy, complex categories imposed on groups in a particular social context. The modular framework presents persemes as easily distinguishable entities chosen independently from one another rather than on the basis of organizing images like sex and gender, as ontologically flat and equivalent rather than constituting the genetically modified person in distinct ways, and as decontextualized rather than as links between family members or as products of collaboration.

In the second half of the article, we considered the normative implications of these assumptions about how persemes exist and examined how the modular framework might have restricted the progress of the HGGM debate. This framework may predispose bioethicists to accept objective list theories of well-being because it implies that persemes correspond to discrete and unconnected aspects of well-being, that designer determinations matter more than the attitudes of the modified person, and that genetically modified people are somewhat artificial. These theories also benefit from the way that hundreds of scholars have framed the debate with references to the brainwashed castes of *Brave New World.* It remains unclear how the objective list should be developed other than through intuition, but participants in the HGGM debate typically prioritize autonomy, favouring capacities over restrictive “life plans” that might rely on connections between persemes or contextual information. Many participants also treat these capacities as fungible economic goods amenable to redistribution. These assumptions fit best with the political philosophy of neutralist liberalism associated with John Rawls and his followers, to the exclusion of perspectives like those of disability advocates who deny the neutrality of persemes like hearing and height.

As a result, participants in the bioethical debate over HGGM may end up talking past one another. The abstraction and mythic sense of importance associated with the debate can push stakeholders toward polarizing bioethical issues, while the modular framework promotes awkward imagery of genetically modified people as technical assemblages. Opponents of HGGM often find themselves arguing over key concepts like the definition and importance of humanness but without the ability to express themselves in a matching ethical vocabulary, placing them in a bind which may further their reliance on narratives from science fiction and religion. Although understanding how modular “building block” rhetoric emerged and how persemes function in the HGGM debate will require further research, this work will ultimately be necessary to help us classify future people and their differences in ways that are more suitable to productive bioethical debate ([393], pp. 52–53).

One approach that might help participants in the HGGM debate to understand each other’s perspectives would be for consequentialist bioethicists to discuss more explicitly how they choose theories of well-being and activities associated with the “good life,” reflecting on how each are linked to different images of genetically modified people and their potential ways of living ([287], p. 271, [340]). Meanwhile, deontologists and virtue ethicists can explain their interpretations of human nature in a more analytical fashion rather than taking humanness as inherently mysterious or beyond rational debate ([25], pp. 65–66, [91], p. 85, [210]). Both groups should endeavour to provide greater clarity concerning key values like autonomy, helping us to understand how they differ between the proponents and opponents of different HGGM applications.

Another approach that could help achieve progress in the debate is for scholars to critique perseme rhetoric and consider alternative conceptual frameworks to the “building blocks” described in this article. This could involve moving away from the perseme mode when possible: for example, when discussing the lives and experiences of genetically modified people, we could move towards the molecular mode and the effects of specific, well-characterized genetic variants [11]. We could also move towards the life plan mode, considering how persemes like sex or strength, as well as concepts like autonomy, achieve definition in the context of future social arrangements and commonly-discussed careers like athletes and super-soldiers.

We could also try to discuss persemes in ways that make them seem less independent, flat, and decontextualized. Erik Parens proposes a “binocular” perspective that would oscillate between seeing genetically modified people as objects and as subjects ([394], pp. 33–46). Rather than conceptualizing future people as technical assemblages of discrete parts, in violation of what we actually know of genetics, we could add a second “conceptual lens” to the HGGM debate by viewing genetically modified people as “more or less integrated bundles of bodies, minds, histories, purposes etc.,” as Alfred Nordmann put it [83]. In parallel, we could try to see human categories less as internal components and more as connections or comparisons between people, or to see them as ways of being within society, similar to the way in which the idea of disabilities as independent entities is rejected in the social model of disability. However, these alternatives might be difficult to represent in language that is as straightforward and compelling to a wide audience as the conventional, science-fiction influenced “boilerplate” of culturally salient components for genetically modified people.

One option for expressing holistic conceptual frameworks in accessible language might involve looking to the HGGM writings of scholars such as Christian theologians, who tend to reject both the idea of persemes as modular components and the idea of genetically modified people as detached individuals. Attention to these anti-reductionist ontologies might provide a thought-provoking contrast to those of mainstream bioethics, and, as theologians served as scientists’ primary challengers in the HGGM debate until the 1980s, might also help us to understand the historical context in which the modular framework emerged (So D: Christian theological "lenses" for the ontology of genetically modified people, submitted).

Efforts to move from persemes to life plans or toward more “integrated” models of future people could be facilitated by adding context outside the simple event schemas of the modular framework and developing more concrete and complete scenarios for future persons’ identity, purpose, and place in society. One of the best efforts to contextualize contemporary visions of HGGM was put forward in the Nuffield Council on Bioethics’ 2018 report *Genome Editing and Human Reproduction* ([86], p.114, 127), which considered genome editing not simply as a decision about the characteristics of future people but as a technology shaped by changing societies. In another study, Selin et al. asked multidisciplinary groups of experts to develop narratives of four futures across the axes of distributed or centralized power and public or market interest [395]. Both projects considered contextual influences on the governance of HGGM like increases in populist nationalism, a factor which, according to Armin Grunwald, has been largely obscured by the libertarian and individualist assumptions of the debate ([396], p.48). However, these efforts have proceeded at a broad scope and high level of abstraction, considering genome editing as a technology within changing social systems rather than examining the way we imagine future people, their creation, and their lives [397].

Richer scenarios about genetically modified people might require input from a wider range of academic disciplines. Indeed, calls for the involvement of experts outside of genetics and bioethics are fairly frequent in the debate ([322, 398], p. 2, [399, 400], p.238). The most common approach is to advocate for the inclusion of social scientists ([295], p. 36, [401]), including anthropologists ([322, 394], p. 36, [400], p.238), sociologists ([125, 398], p. 2, [400], p.238), psychologists ([64], p. 110–117, [256], p. 186, [400], p.238), and STS scholars [402]. Some scholars argue that these disciplines can help us envision HGGM more realistically than previous contributors to the debate have done [403]. Jay Clayton observes that bioethicists use science fiction-like speculation rather than projecting the future based on methodically gathered data [125]; similarly, Allen Buchanan accused opponents of enhancement like Kass, Sandel, and Habermas of representing “one of the last academic strongholds of a priori psychology and sociology” ([197], pp. 5, 9). Better data about the possible experiences and societal implications of genetically modified people might be gathered through empirical research, using methodological tools and standards from the social sciences ([40, 60], p. 303, [400], p.238, [403]).

Other participants in the HGGM debate have suggested bringing in experts from the arts, such as novelists, filmmakers ([402, 404, 405] 2:38), and critics [125, 283]. This could help us to understand the influence of artistic representations of HGGM on current stakeholders ([405] 2:38), generate possible scenarios of the future ([406] p. 162), and reconnect the abstract debate to actual human experiences ([394], p. 36). Literary representations can be a particularly good way of exploring the embeddedness of human categories in a world where subjects are materially and socially connected to one another ([118], p. 189, [407], p. 10). In contrast to the simple assembly of modular building blocks, methods from the arts could help us to imagine HGGM as a process that involves the interaction of numerous actors, not least the active agency of genetically modified people as they grow and develop within future societies ([118], p. 189, [123]).

Since science fiction is already heavily involved in the HGGM debate as a framing device, it might be possible to enliven it from dead metaphor into a useful method of envisioning possible futures. As we proposed in a previous article, [120] this could involve curating scenarios from pre-existing science fiction works about HGGM or recruiting contemporary science fiction authors as active participants in conferences, workshops, and organizations involved in public engagement and governance. A similar approach might consist of helping current stakeholders to explore and elaborate their views by writing fiction [397]. Narrative contributions to the debate can help bring together technological, ethical and social science expertise that often remains separate in HGGM reports [395]. Narratives can also contribute concreteness by making social and technological changes vivid and graspable at the human scale ([53], p.94, [146, 397]) and by dramatizing elements from stakeholders’ real-life experiences of enhancements and emerging reproductive procedures.

In such efforts to expand and enrich our images of genetically modified people, it will be important to consider whether mainstream ethical approaches to HGGM are rooted in American culture, as scholars like John H. Evans, Leon Kass, and Diane B. Paul have suggested ([30], pp. 12, 130, [32], p. 144, [50, 322]). Stakeholders outside the Anglosphere might have significantly different conceptual frameworks: there is some evidence that East Asian subjects in social psychology experiments endorse fewer beliefs associated with biological essentialism [177, 180] and more frequently describe themselves by social roles rather than traits [408]. If so, the HGGM debate’s lean towards discussing persemes over life plans may not be equally present for stakeholders whose experiences were not shaped by English-language scholarship and by the Anglo-American eugenics movement which formed the backdrop for the first stages of the debate. Continuing engagement efforts at the international level may reveal an even broader range of visions for genetically modified people and provide another incentive to continue the expansion of the HGGM debate.

As the fears and hopes associated with emerging biotechnologies become increasingly powerful motivations for societal action, it seems clear that many scholars will continue to focus their energies on speculative bioethics. More likely than not, these bioethicists will continue to rely largely on the discipline’s traditional tools for conceptual analysis. However, these approaches may be insufficient to resolve opposing stakeholders’ tendencies to talk past each other, especially when unresolved schisms leave space for science fiction, myth, and religion as motivators and modes of participation in the HGGM debate. This article argued that one step forward for the debate might involve interdisciplinary research into the foundational assumptions that produced the modular framework for discussions of genetically modified people, including the historical context in which it emerged and the psychology that underpins its function in the debate. These avenues of research allow us not just to pinpoint how conventional ethical arguments function but to imagine how these discourses might have been otherwise and to provide the tools needed to envision alternate paths for the HGGM debate going forward. Hopefully, the findings presented in this article will help to make these opportunities more accessible for scholars both within bioethics and in adjoining disciplines and stakeholder groups, whose involvement is likely to be crucial in the evolving debate.
